# Sex-Specific Fatigue and Muscle Activation Responses During Single-Leg Side-Hop and Pelvic Stability Assessments Among Active Young Adults

**DOI:** 10.3390/sports14020056

**Published:** 2026-02-03

**Authors:** Emilija Stojanović, Oliver Faude, Alexander Ferrauti, Dragan Radovanović, Aaron T. Scanlan, Ralf Roth

**Affiliations:** 1 Department of Physiology, Faculty of Medical Sciences, University of Kragujevac, 34000 Kragujevac, Serbia; stojanovic.emilija@yahoo.com; 2 Department of Training and Exercise Science, Faculty of Sport Science, Ruhr University Bochum, 44801 Bochum, Germany; alexander.ferrauti@ruhr-uni-bochum.de; 3 Department of Sport, Exercise and Health, University of Basel, 4052 Basel, Switzerland; oliver.faude@unibas.ch; 4 Faculty of Sport and Physical Education, University of Niš, 18000 Niš, Serbia; fiziologija@fsfv.ni.ac.rs; 5 School of Health, Medical and Applied Sciences, Central Queensland University, Rockhampton, QLD 4700, Australia; a.scanlan@cqu.edu.au; 6 S.P.O.R.T. Research Cluster, Central Queensland University, Rockhampton, QLD 4700, Australia

**Keywords:** male, female, electromyography, injury, landing strategy, stabilization

## Abstract

This study examined fatigue- (within-group) and sex-related (between-group) differences in physical performance and muscle activation during physical assessments. Physically active college students (20 males, 20 females) completed side-hop and pelvic stability tests after a warm-up (T-1) and mobilization exercises (T0) and then following each with five 8 min runs at 70% of individualized peak velocity as the fatiguing protocol (T1–T5). No significant within-group performance differences were observed across tests (T0–T5). However, males completed more hops (*p* < 0.001) and had shorter ground contacts (*p* < 0.05) than females in the side-hop test with no significant sex-based stability differences. Electromyography data revealed reduced activation (*p* < 0.05) in various muscles (gastrocnemius, vastus medialis, biceps femoris, gluteus medius, gluteus maximus, erector spinae, obliquus abdominis) under fatigue (various timepoints between T1–T5) compared to baseline (T-1) across tests. Males displayed greater relative reductions in activation (*p* < 0.01) from pre-activation to ground contact in the gastrocnemius and biceps femoris during the side-hop test. Females exhibited reduced vastus medialis (*p* = 0.02) activation during the side-hop test and reduced biceps femoris (*p* = 0.04) activation during the pelvic stability test than males. Physical performance remained stable under fatigue, with sex-specific muscle recruitment strategies appearing as possible compensatory mechanisms.

## 1. Introduction

Those participating in sport frequently experience intermittent high-intensity demands during training and competition, including cutting, sharp decelerations, pivots, and landing maneuvers—common situations in which anterior cruciate ligament (ACL) injuries occur [1]. ACL injury is often regarded as one of the most devastating sports injuries, with rehabilitation after reconstruction typically lasting 6–12 months. ACL injury incidence is typically greatest among young adults, coinciding with sports participation and undertaking high physical demands [2]. In addition to age-specific patterns, anatomical differences between sexes underpin a higher risk of sustaining an ACL injury among females, including increased tibial and meniscal slopes [3,4], narrower femoral notches [3], smaller ACL [3,5] and a greater Q angle [6].

Beyond anatomical and sex-related factors, muscle fatigue has also been implicated as a contributing factor to ACL injury risk, as it alters lower-limb biomechanics during sporting maneuvers, often resulting in increased lateral knee loading—characterized by excessive valgus and internal tibial rotation—which places greater strain on the ACL and compromises joint stability [7]. This concern is reinforced by evidence demonstrating that most lower-limb injuries occur in the later stages of activity and sporting competition [8,9,10], highlighting the potential progressive deterioration of neuromuscular control with fatigue. Supporting this notion, muscle fatigue has been shown to increase knee joint laxity [11], reduce balance [12], impair proprioception [13] and diminish motor control [14,15]. The cumulative effects of muscle fatigue may therefore disrupt lower-limb movement strategies and heighten injury susceptibility during different sport activities [16].

While muscle fatigue appears to influence certain biomechanical and neuromuscular aspects, inconsistent results concerning muscle activation under fatigued conditions have limited the available understanding of muscle responses relevant to ACL injury prevention. In this regard, the hamstring muscles play a key role in dynamic knee stability as critical ACL antagonists. Research examining hamstring muscle activation following different physical fatigue protocols has yielded equivocal outcomes, reporting unchanged [17,18,19], reduced [20,21,22,23,24,25], or even increased [26,27] activation levels following the fatigue stimuli. These discrepancies in findings may arise from the array of fatigue protocols (step-ups [17], Wingate anaerobic test [18], vertical jumps [19], intermittent exercises [20], box jumps [21], squatting motions [22], incremental running with jumps [23], sub-maximal leg press exercise [24], bodyweight squats [25], knee flexion [26], squats, jumps, and steps [27]), as well as task execution, such as single-leg (horizontal hop [17], side-cutting [20], drop jump landings [18,26,27]) versus double-leg activities (drop jump landings [19,21,24,25], countermovement jump [21], hopping [22] and forward-side jump [23]). Studies have also documented inconsistent results regarding sex-related differences in hamstring activation when under fatigue, with some reporting similar activation between sexes [21,22,25], while others found delayed activation [24] or greater activation in females compared to males [27]. Taken together, these inconsistencies highlight the need for future research analyzing muscle responses under relevant tasks which closely simulate conditions associated with ACL injury, such as single-leg landing [1], to better understand hamstring activation in high-risk situations.

In addition to the hamstring muscles, the vastus medialis and gastrocnemius muscles play key roles in knee stabilization and dynamic joint control during landing activities [28]. Consequently, understanding activation changes in these muscles in response to physical fatigue may be useful in ascertaining injury risk and associated mechanisms during landing activities. Previous studies have reported variable activation responses in the vastus medialis [either unchanged [20,24], reduced [21], or increased activation [17,26] and gastrocnemius [unchanged [17,19], reduced [20,21,24], or increased activation [22,26] following fatigue protocols. Although not all fatigue protocols directly replicate the specific demands associated with ACL injury, these collective findings may reflect muscle-specific neuromuscular resilience that could influence joint loading patterns and injury risk under fatigued conditions. In this way, quantifying activation of wider stabilizing muscles—such as the gluteus maximus, gluteus medius, erector spinae, and obliquus abdominis—should facilitate broader insight into trunk-pelvis control strategies that affect lower-limb mechanics relevant to ACL injury risk [29].

Given the variability reported in the literature concerning fatigue-related muscle activation responses across different muscles and between sexes, more research is needed to develop a definitive consensus on neuromuscular strategies relevant to ACL injury risk. Measuring muscle activation during a single-leg side-hop task is particularly relevant, as it reflects a high-risk single-leg landing maneuver commonly associated with ACL injury. Moreover, evaluating pelvic stability may provide further valuable insight into muscle activation responses within trunk and hip stabilizers [30], which plays an important role in knee joint loading and ACL injury risk. Therefore, the aim of this study was to examine fatigue- (within-group) and sex-related (between-group) differences in physical performance (lower-limb function and pelvic stability) and muscle activation responses relevant to ACL injury risk.

## 2. Materials and Methods

### 2.1. Design

**Figure 1:**
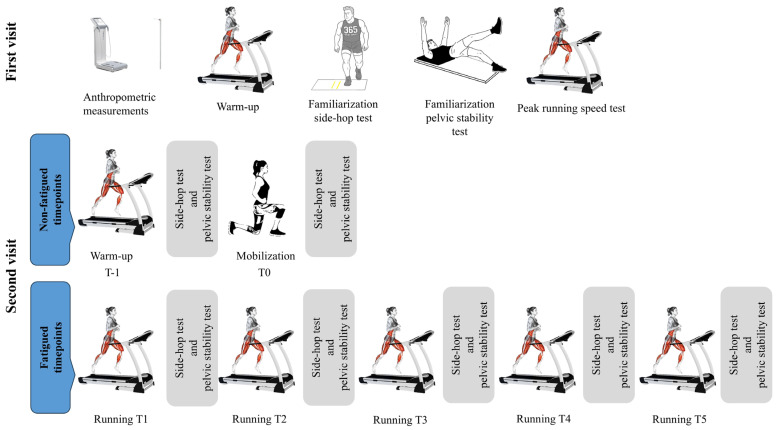
A schematic illustration of the study design.

A cross-sectional experimental design was employed, with each participant attending two testing sessions separated by 5–7 days. A schematic illustration of the study design is shown in Figure 1.

### 2.2. Participants

Trained Tier 2 male (n = 20) and female (n = 20) athletes (engaged in various sports) attending a Swiss university volunteered to participate in this study. Participants had to be partaking in training volume at least 4.5 h·wk^−1^ (~3 times per week) for the past 6 months [29], and free from injury and any symptoms affecting the lower limbs, trunk, or ability to perform physical activity over the last 12 months prior to participation in the study. Participants were given full details of the study procedures, risks, and benefits before providing written consent to participate. This study was approved by the regional ethics committee (Ethikkommission Nordwest- und Zentralschweiz, ID: 2020-00130), with all procedures conducted in accordance with the Declaration of Helsinki.

### 2.3. Procedures

During the first session, participants completed anthropometric assessment, a running-based warm-up, familiarization trials for the side-hop test and pelvic stability test, and a peak running speed test. Body mass and composition were determined for each participant using a multifrequency bioelectrical impedance analyzer (InBody 720; Biospace Co., Ltd., Seoul, Republic of Korea) following standard measurement procedures [31]. The validity (*r* = 0.90) and reliability (intraclass correlation coefficient (ICC) = 0.98, standard error of measurement [SEM] = 0.99) of the InBody 720 for measuring body fat composition have been supported previously [31]. Height was measured using a portable stadiometer (Seca 220; Seca Corporation, Hamburg, Germany) with a graduation of 0.1 cm. The peak running speed test was completed on a motorized treadmill (Zebris FDM-T, Zebris medical GmbH, Isny, Germany) at a grade of 3°. The test started at a speed of 8 km⸱h^−1^ with constant speed increases of 1 km⸱h^−1^ each minute until exhaustion was reached [32]. Peak running speed (V_peak_) was taken when maximal effort during the test was deemed to be achieved, with the following criteria being met [33]: (1) heart rate ≥100% of age-predicted maximum heart rate (HR_max_) using the age-based equation (220 − age); and (2) rating of perceived exertion (RPE) was ≥9 using Borg’s Category-Ratio 10 scale.

During the second session, participants underwent seven separate trials of the side-hop test and pelvic stability test before, and interspersed throughout, a fatiguing running protocol. In this regard, tests were administered after the walking-based warm-up (T-1), after further mobilization exercises and before the first bout of the fatiguing protocol (T0), and then after each of five running bouts as part of the fatiguing protocol (T1–T5). The walking-based warm-up was completed on the motorized treadmill at 6 km∙h^−1^ for 5 min, while an 8 min active recovery consisting of mobilization and preparation exercises (10–15 hops) was conducted between the first two testing timepoints. The fatigue protocol consisted of five 8 min treadmill-based running bouts at 70% of individualized V_peak_, with each bout separated by 3–5 min. Testing alongside passive standing recovery was administered in each of these periods between running bouts (35). The variability in target speed was ±0.5 km⸱h^−1^ during the last two or three running bouts in the protocol, which was adjusted as necessary (if participants were unable to complete the protocol) and has been supported previously [34]. All testing was conducted by the same two experienced researchers with heart rate continuously recorded throughout tests using an Acentas Team System (Acentas, Hörgertshausen, Germany). 

#### 2.3.1. Side-Hop Test

The side-hop test was performed using a dual force plate system (Kistler Type 9286BA and 9286AA, Kistler Group, Winterthur, Switzerland), whereby two yellow adhesive strips of tape were placed parallel, 30 cm apart, on the inner edge of both plates. The setup adopted during the side-hop test is shown in Appendix A.1. Participants stood on the test leg (right leg on the left plate or left leg on the right plate) and, in response to the tester command (“Ready—Ready—Go”), hopped laterally on the same leg from one force plate to the other past the yellow strips as quickly as possible for 10 s [35]. Both legs were separately tested (commencing with the left leg on all occasions) with the arms free to move throughout. If a participant made foot contact with a yellow strip, the hop was not counted. The goal was to generate as many valid attempts as possible. Testing was performed wearing standardized athletic shoes (Imviso 100, Kipsta, France), with the test timed by one researcher using a stopwatch while the other researcher recorded performance. Test performance was assessed as the number of total hops and valid hops, with the average ground contact time (s) across all hops also taken. This hopping task was selected due to epidemiological injury data indicating that knee injuries, particularly ACL injuries, frequently occur during single-leg jump landings [36]. Analyses between the non-fatigued testing timepoints (T-1 and T0) in this study revealed relatively strong reliability for the number of valid hops (ICC = 0.97, SEM = 0.73 hops).

#### 2.3.2. Pelvic Stability Test

The pelvic stability test is a modified version of the core-oriented “deadbug bridging (DBB) performance test,” which has been used to assess posterior chain stabilization in athletes [37]. A core-oriented DBB test was modified to increase instability and enhance hip muscle involvement, ensuring greater specificity in the pelvic stability test protocol [38]. The movement patterns performed in this test are shown in Appendix A.2. Participants began the test in a supine position on the floor, with their legs hip-width apart and shoulders flexed to 90°. Participants were then instructed to lift the pelvis and one leg from the floor, creating a unilateral bridge position, held for 3 s. The leg was then returned to the starting position before the movement was repeated with the other leg. Three trials for each leg were performed without the hips touching the floor. The maximum hip drop displacement (cm) was determined with a Vicon camera system (version 2.7.1, Vicon Motion Systems Ltd., Oxford, UK) and taken as the outcome measure. Eight high-speed cameras were utilized to provide extensive coverage in measuring hip drop displacement. Each leg was tested separately as the supporting leg, with the position of the marker on the opposite hip (located on the anterior superior iliac spine) analyzed for movement. Analyses between the non-fatigued testing timepoints (T-1 and T0) in this study revealed relatively strong reliability for hip drop displacement in this test (ICC = 0.75, SEM = 4.6 cm).

#### 2.3.3. Muscle Activity

Muscle activity was assessed during both the side-hop and pelvic stability tests on the right leg only using a Myon 320 electromyography (EMG) system (Myon AG, Schwarzenberg, Switzerland). Intra-session and inter-day reliability of the Myon 320 EMG have been documented previously (ICC = 0.63–0.97, SEM = 1.49–2.32 mV, coefficient of variation [CV] = 4.7–7.2%) [39]. Participants were instrumented with seven pairs of EMG electrodes, each with a diameter of 34 mm (Ambu^®^ BlueSensor P, Ambu GmbH, Bad Nauheim, Germany). Silver/silver-chloride (Ag/AgCl) commercially available gel electrodes were used for recording EMG activity [40]. Standard EMG skin preparation methods were utilized including shaving and lightly abrading the skin, then wiping with alcohol to reduce electrical impedance. The electrodes were positioned on the right side via anatomical surface landmarks located by palpation on the belly of each muscle [24] to record activity of gastrocnemius (caput mediale), vastus medialis, biceps femoris (long head), gluteus medius, gluteus maximus, erector spinae, and obliquus externus abdominis. Sensor placement followed SENIAM (Surface ElectroMyoGraphy for the Non-Invasive Assessment of Muscles) guidelines and was aligned with the expected fiber orientation of each muscle. Due to the high-impact landings during side-hops, the EMG transmitter boxes and electrodes were fixed and secured with adhesive tape to ensure consistent contact with the skin, as shown in Appendix A.3. Muscle activity was analyzed in the pre-activation phase set 150 ms before landing [41,42], and during ground contact immediately upon landing in the side-hop test. EMG data were processed using Matlab (R2024b, MathWorks, Natick, MA, USA). Representative raw and processed EMG recordings from one participant during the side-hop and pelvic stability assessments at T1 are provided in Appendix A.4.

### 2.4. Statistical Analyses

An a priori power analysis using G*power software (version 3.1.9.4; Heinrich Heine University Düsseldorf, Düsseldorf, Germany) indicated a required sample size of 18 participants (*p* = 0.05, effect size [ES] = 0.25; power = 0.80), using an effect estimate from research examining sex-related differences in hamstring muscle activation following a fatigue protocol [25]. EMG data were expressed as a percentage change relative to baseline (taken at the T-1 testing timepoint) individually for each participant across all timepoints. Normality of all data was confirmed with the Shapiro–Wilk test. Consequently, data were descriptively determined as mean ± standard deviation (SD).

Performance differences were determined using mixed 7 × 2 analyses of variance (ANOVAs) with one within-participants factor (time: 7 timepoints from T-1–T5) and one between-participants factor (sex). EMG differences (for each muscle) were determined using mixed 6 × 2 × 2 ANOVAs [with two within-participants factors (time: 6 timepoints from T0–T5 and phase: pre-activation and ground contact) and one between-participant factor (sex)] during the side-hop test, and mixed 6 × 2 ANOVAs [with one within-participants factor (time: 6 timepoints from T0–T5) and one between-participants factor (sex)] during the pelvic stability test. Partial eta-squared ($\eta_{p}^{2}$) was determined to indicate the effect size (ES) for each ANOVA with values interpreted as: no effect ($\eta_{p}^{2}$ < 0.01), small effect (0.01 < $\eta_{p}^{2}$ < 0.06), medium effect (0.06 < $\eta_{p}^{2}$ < 0.14) and large effect ($\eta_{p}^{2}$ > 0.14) [43]. When significant effects were observed, within-group changes between timepoints were assessed using Bonferroni post hoc testing. Statistical significance was reached when *p* < 0.05 with all analyses conducted using IBM SPSS software (version 19; IBM Corp., Armonk, NY, USA).

## 3. Results

### 3.1. Participant Characteristics

Participant characteristics are displayed in Table 1.

### 3.2. Fatigue Protocol Demands

**Figure 2:**
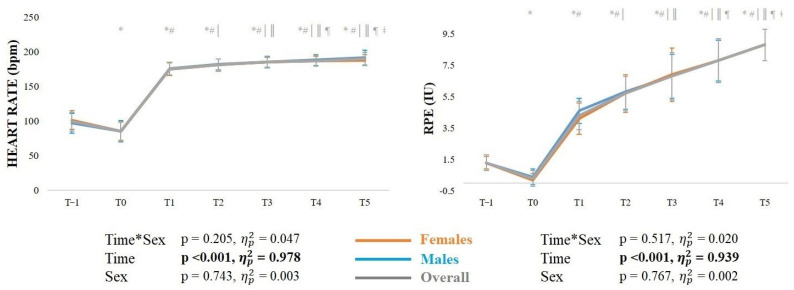
Mean ± standard deviation heart rate and rating of perceived exertion (RPE) across timepoints. *Note*: * significantly (*p* < 0.05) different from T-1; # significantly (*p* < 0.05) different from T0; | significantly (*p* < 0.05) different from T1; ‖ significantly (*p* < 0.05) different from T2; ¶ significantly (*p* < 0.05) different from T3; ǂ significantly (*p* < 0.05) different from T4.

The mean ± SD heart rate (HR) and RPE responses during each running bout along with outcomes from the corresponding ANOVAs are shown in Figure 2. There was a significant main effect of time in both heart rate and RPE, with progressive increases (*p* < 0.001) across bouts.

### 3.3. Side-Hop and Pelvic Stability Test Performance

The outcomes of the ANOVAs for the side-hop and pelvic stability tests are presented in Table 2, with the mean ± SD for outcomes from these tests presented in Table 3. Regarding total hops in the side-hop test, there were significant main effects of time for both legs, with less hops performed at T-1 compared to all other timepoints. There were also significant main effects of sex for both legs, whereby males completed more total hops than females. For valid hops, there was a significant main effect of time for the right leg, with less performed at T-1 compared to T0 and T1. Regarding ground contact time, there was a significant time*sex interaction for the left leg. In turn, there was a significant main effect of time for the right leg, with longer contacts at T-1 compared to all other timepoints. There were also significant main effects of sex for both legs, with males having shorter contacts than females. No significant interactions or main effects in hip drop displacement were evident for both legs in the pelvic stability test.

### 3.4. Muscle Activation During the Side-Hop Test

**Figure 3:**
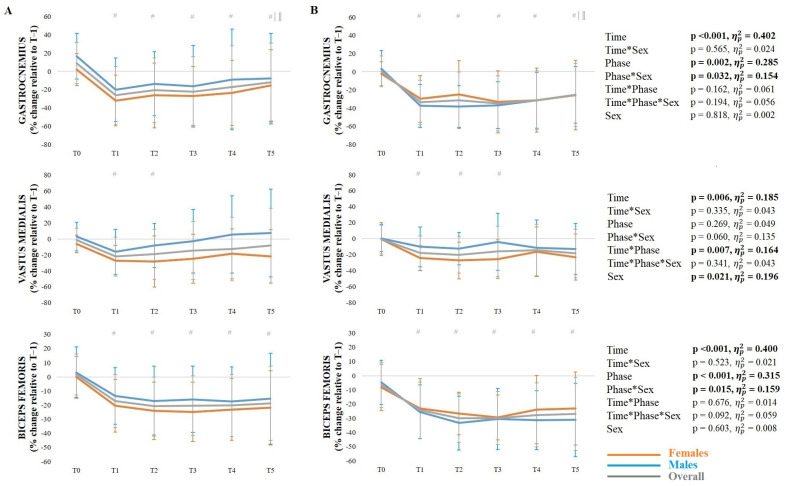
Mean ± standard deviation muscle activity (gastrocnemius, vastus medialis and biceps femoris) during the side-hop test in the pre-activation phase (**A**) and ground contact phase (**B**). *Note*: # significantly (*p* < 0.05) different from T0; | significantly (*p* < 0.05) different from T1; ‖ significantly (*p* < 0.05) different from T2.

**Figure 4:**
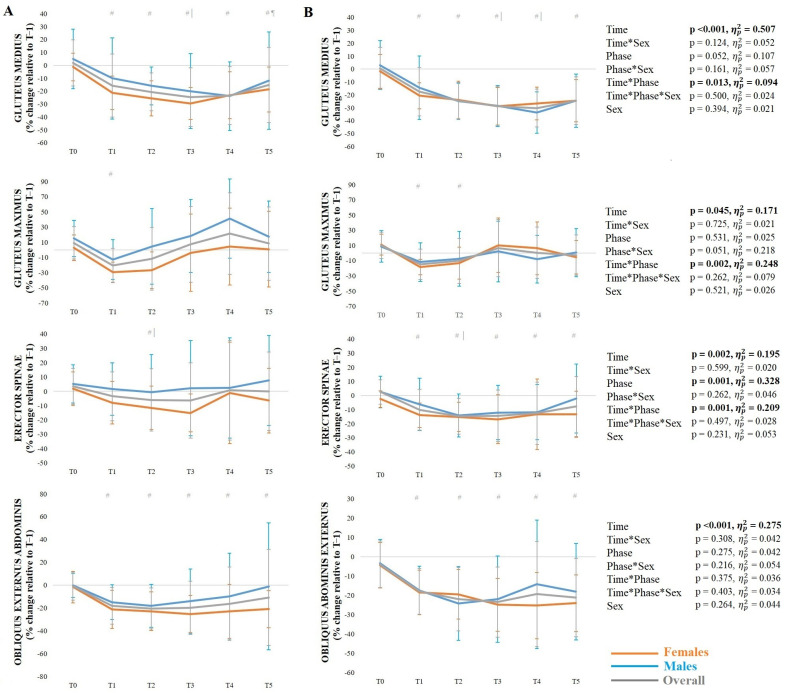
Mean ± standard deviation muscle activity (gluteus medius, gluteus maximus, erector spinae, and obliquus externus abdominis) during the side-hop test in the pre-activation phase (**A**) and ground contact phase (**B**). *Note*: # significantly (*p* < 0.05) different from T0; | significantly (*p* < 0.05) different from T1; ¶ significantly (*p* < 0.05) different from T3.

The mean ± SD muscle activity during the side-hop test at the pre-activation (A) and ground contact phase (B) across timepoints, along with the results of the corresponding ANOVAs, are presented in Figure 3 and Figure 4. Regarding the gastrocnemius, there was a significant phase*sex interaction, with a greater change (%) from the pre-activation phase to the ground contact phase in males than females (*p* = 0.008). There was also a significant main effect of time, with reduced activation at T1–T5 compared to T0 (*p* < 0.001), as well as at T5 compared to T1 (*p* = 0.035) and T2 (*p* = 0.005). For the vastus medialis, there was a significant time*phase interaction, with reduced activation during the pre-activation phase at T1 (*p* = 0.001) and T2 (*p* = 0.004) compared to T0, as well as during the ground contact phase at T1–T3 compared to T0 (*p* = 0.001–0.022). In addition, there was a significant main effect of sex, with females demonstrating reduced activation than males. Regarding the biceps femoris, there was a significant phase*sex interaction, with a greater change (%) from the pre-activation phase to the ground contact phase in males than females (*p* = 0.008). There was also a significant main effect of time, with reduced activation at T1–T5 compared to T0 (*p* < 0.001).

For the gluteus medius, there was a significant time*phase interaction, with reduced activation in the pre-activation phase at T1–T5 compared to T0 (*p* ≤ 0.001–0.002) and at T3 compared to T1 (*p* = 0.002) and T5 (*p* = 0.010), as well as during the ground contact phase at T1–T5 compared to T0 (*p* = < 0.001) and at T3 and T4 compared to T1 (*p* < 0.001). Regarding the gluteus maximus, there was a significant time*phase interaction, with reduced activation in the pre-activation phase at T1 compared to T0 (*p* < 0.001), as well as during the ground contact phase at T1 (*p* = 0.001) and T2 (*p* = 0.004) compared to T0. For the erector spinae, there was a significant time*phase interaction, with reduced activation in the pre-activation phase at T2 compared to T0 (*p* = 0.002) and T1 (*p* = 0.030), and during the ground contact phase at T1–T5 compared to T0 (*p* < 0.001), and at T2 compared to T1 (*p* = 0.029). Regarding the obliquus abdominis externus, there was a significant main effect of time, with reduced activation at T1–T5 compared to T0 (*p* ≤ 0.001–0.005).

### 3.5. Muscle Activation During the Pelvic Stability Test

**Figure 5:**
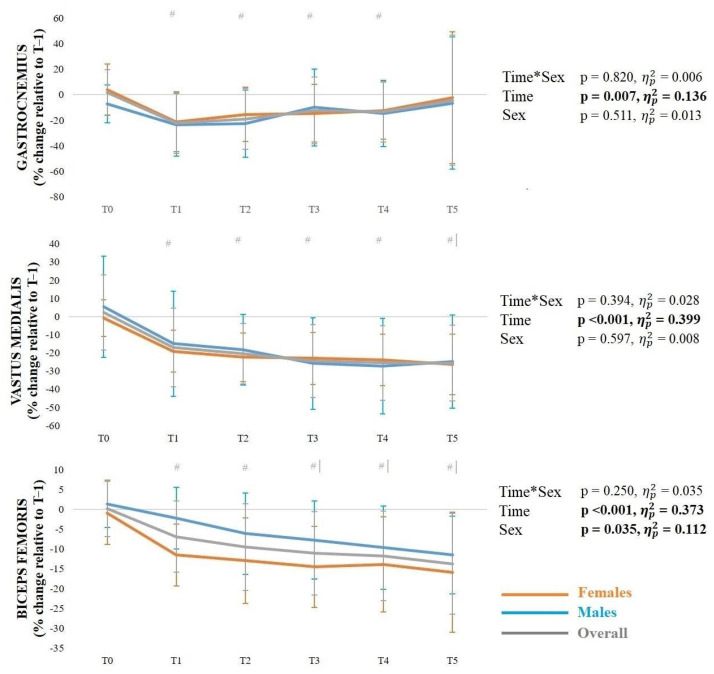
Mean ± standard deviation muscle activity (gastrocnemius, vastus medialis and biceps femoris) during the pelvic stability test. *Note*: # significantly (*p* < 0.05) different from T0; | significantly (*p* < 0.05) different from T1.

**Figure 6:**
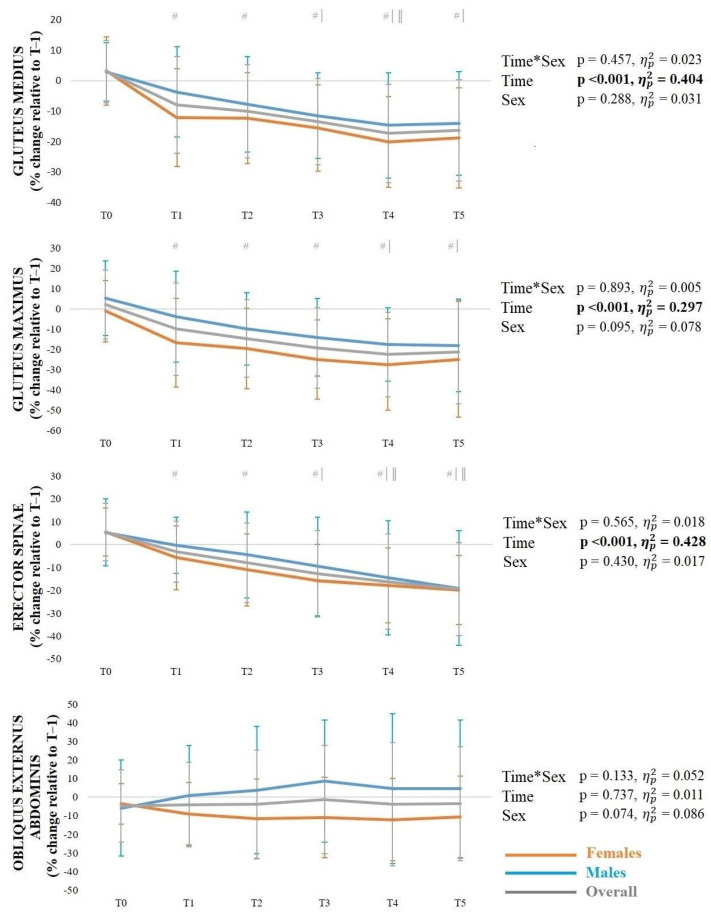
Mean ± standard deviation muscle activity (gluteus medius, gluteus maximus, erector spinae and obliquus externus abdominis) during the pelvic stability test. *Note*: # significantly (*p* < 0.05) different from T0; | significantly (*p* < 0.05) different from T1; ‖ significantly (*p* < 0.05) different from T2.

The mean ± SD of muscle activity during the pelvic stability test across timepoints, along with the results of the corresponding ANOVAs, are presented in Figure 5 and Figure 6. Regarding the gastrocnemius, there was a significant main effect of time with reduced activation at T1–T4 compared to T0 (*p* ≤ 0.001–0.02). For the vastus medialis, there was also a significant main effect of time, with reduced activation at T1–T5 compared to T0 (*p* < 0.001), as well as at T5 compared to T1 (*p* = 0.01). Considering the biceps femoris, there was a significant main effect of time, with reduced activation at T1–T5 compared to T0 (*p* < 0.001), as well as at T3–T5 compared to T1 (*p* = 0.01–0.03). There was also a significant main effect of sex, with males having less reduction in activation than females.

For the gluteus medius, there was a significant main effect of time, with reduced activation at T1–T5 compared to T0 (*p* < 0.001), at T3–T5 compared to T1 (*p* = 0.002–0.03), and at T4 compared to T2 (*p* = 0.002). Regarding the gluteus maximus, there was a significant main effect of time, with reduced activation at T1–T5 compared to T0 (*p* ≤ 0.001–0.02), as well as at T4 (*p* = 0.02) and T5 (*p* = 0.05) compared to T1. Considering the erector spinae, there was a significant main effect of time, with reduced activation at T1–T5 compared to T0 (*p* < 0.001), at T3–T5 compared to T1 (*p* ≤ 0.001–0.003), and T4 (*p* = 0.01) and T5 (*p* < 0.001) compared to T2.

## 4. Discussion

The major findings of this study demonstrate that muscular fatigue induced by repeated running stimuli alters landing strategy in a sex-specific manner, as reflected by changes in lower-limb muscle activation. More precisely, reduced activation of the gastrocnemius, vastus medialis, biceps femoris, gluteus medius, erector spinae, and obliquus abdominis externus were evident across the running-based fatiguing protocol in both males and females. In turn, males demonstrated greater reliance on the vastus medialis, with task-specific activation observed across different muscles to maintain performance in each sex. Despite the observed compensatory strategies adopted across the fatiguing protocol, the reduced EMG amplitudes indicate altered neuromuscular coordination, potentially compromising joint stability.

While muscular fatigue was observed, performance outcomes during the side-hop and pelvic stability tests remained unchanged throughout the fatiguing protocol. In contrast to these findings, Lesinski et al. [21] reported a reduction in drop jump and countermovement jump height following a fatiguing protocol that involved consecutive, repetitive vertical double-leg box jumps until failure. The discrepancy between studies may be attributed to differences in fatigue protocols and task demands, making direct comparisons difficult. Interestingly, although no decline in physical performance was evident in our study, we observed consistent reductions in EMG amplitudes across multiple lower-limb and trunk muscles. Typically, muscle fatigue is expected to result in increased EMG amplitude [44,45] due to the recruitment of additional motor units to sustain force production. However, the reduced EMG activity we observed may reflect an altered—and potentially less effective—motor control strategy or shifts in muscle coordination patterns that minimize neuromuscular demands while preserving performance. Although such compensatory mechanisms may be sufficient to maintain task execution, they could compromise joint stability and neuromuscular control, particularly under fatigued conditions. This response may increase the risk of injury, as optimal muscle activation is essential for maintaining movement quality and protecting musculoskeletal structures [46].

We observed similar trends in activation levels for the gastrocnemius, vastus medialis, biceps femoris, gluteus medius, and obliques abdominis externus, with a decreased contractile capability evident during the side-hop test over the course of the fatigue protocol. Our findings are consistent with previous studies demonstrating significantly reduced activation of the hamstrings muscles [20,21,22,23,24,25] and gastrocnemius [20,21,24] following various fatigue protocols. Such reductions have been reported during both single-leg tasks (e.g., side-cutting maneuvers [20]) and double-leg tasks (e.g., drop jump landings [21,24,25], countermovement jumps [21], hopping in place [22], and forward-side jumps [23]). Although the hamstrings and gastrocnemius are not primary agonists for generating movement during landings (such as that in the side-hop test we utilized), they play crucial synergistic and stabilizing roles, particularly in joint stabilization and controlling landing mechanics [47]. The reduced activation of these muscles when functioning as co-agonists or stabilizers may manifest via central fatigue mechanisms [48]. Importantly, decreased hamstring coactivation relative to quadriceps activation could impair knee joint stability, potentially increasing anterior tibial translation and elevating the risk of ACL loading and injury during sport-specific tasks [49].

Our study revealed that males exhibited a less pronounced decline in vastus medialis activity during the side-hop test throughout the fatiguing protocol, with comparable reductions in the gastrocnemius, biceps femoris, gluteus medius, erector spinae, and obliquus externus abdominis in both sexes. The ability of males to sustain vastus medialis activation is particularly important due to its key role in medial knee stabilization, which may contribute to better lower-limb control and movement efficiency, potentially reducing the likelihood of knee injuries. In contrast, inadequate activation of this muscle could lead to excessive knee valgus, a well-known risk factor for ACL injuries [50]. In addition to the vastus medialis, a significant phase*sex interaction was observed in the biceps femoris activation, with males demonstrating a greater reduction from the pre-activation to the ground contact phase than females, likely due to their higher pre-activation levels. This finding highlights sex-related differences in hamstring recruitment strategies when under fatigue. In this regard, sex-related comparisons in muscle activation responses to fatigue in past studies have produced inconsistent results, with some reporting similar reductions in hamstring activation between sexes during the precontact [22,25] or ground contact phase [21,22], while others found delayed [24] or greater hamstring activation (during the ground contact phase) [27] in females compared to males. This diminished hamstring pre-activation we observed in females could potentially limit their ability to mitigate anterior tibial translation and ACL loading. Additionally, males exhibited an increase in gluteus maximus pre-activation at multiple timepoints during the fatiguing protocol (with a phase*sex interaction approaching significance, *p* = 0.051). This trend may indicate a neuromuscular strategy aimed at enhancing joint stabilization before landing. Altogether, these findings suggest that males may rely on greater vastus medialis activity to counteract fatigue-induced reductions in other stabilizing muscles, while also exhibiting a tendency toward increased gluteus maximus pre-activation. In this way, sustained activation of the gluteus maximus muscle may contribute to better neuromuscular control and delay performance decline in males, given its role in knee stabilization and quadriceps force distribution. These findings highlight potential sex-based differences in neuromuscular responses to fatigue, which could inform targeted training and injury prevention strategies.

In addition to sex-based fatigue responses, our results suggest task-specific compensatory strategies are adopted to maintain performance under fatigue. Specifically, the observed muscle activation patterns varied across tasks, with different muscles playing a more prominent role depending on the nature of the test. While a less pronounced reduction in vastus medialis activity was observed during the side-hop test under fatigue in males, a similar neuromuscular strategy was evident in the pelvic stability test, where a smaller reduction in biceps femoris activity was evident in males compared to females. These trends may emerge from the distinct neuromuscular requirements of each task, with vastus medialis activation crucial for controlling knee alignment and absorbing impact in the side-hop test, while hamstring activation supports hip extension and lateral pelvic stability in the pelvic stability test. Altogether, males appear to better preserve activation of these key stabilizing muscles under fatigue, reflecting task-specific compensatory strategies to maintain joint and trunk control.

Despite the novel findings presented in the study, some limitations should be acknowledged. First, although our fatigue protocol elicited high physiological and perceptual intensities indicative of sports activities, it was conducted using treadmill-based running. Consequently, other field-based tests involving specific multidirectional movement stimuli should be implemented to induce fatigue via the muscle recruitment patterns likely adopted when participating in sport. Second, the lack of kinematic data restricts our ability to fully understand fatigue-related changes in movement quality and joint mechanics. Third, activation in other key muscles (e.g., vastus lateralis, rectus femoris, tibialis anterior, soleus, quadratus lumborum) and ground reaction forces were not measured in our study (due to the complexity and duration of the protocol), which limits a more comprehensive understanding of compensatory strategies from being generated. Fourth, although EMG data were individualized as a percentage change relative to the baseline, further research is encouraged to normalize these data to maximum voluntary contractions for more precise comparisons across participants and conditions. Fifth, while EMG data were collected during two specific phases of the side-hop test, further investigation across various timings is encouraged to better understand muscle response in different landing phases.

## 5. Conclusions

In conclusion, this study highlights the significant impact of muscular fatigue on lower-limb muscle activation patterns without affecting performance. While both males and females exhibited reduced activation across the fatiguing protocol in key stabilizing muscles such as the gastrocnemius, vastus medialis, biceps femoris, and gluteus medius, males demonstrated a greater reliance on the vastus medialis to maintain performance when under fatigue. This compensatory strategy, along with the task-specific responses observed, may contribute to better lower-limb control in males. On the other hand, less sustained muscle activation in females may reflect an altered—and potentially less effective—motor control strategy that limits their ability to safely manage knee and hip loading.

## Figures and Tables

**Table 1:** Participant characteristics.

Outcomes	Males (n = 20)	Females (n = 20)
Age (years)	24.8 ± 3.2	24.4 ± 3.0
Height (cm)	179.9 ± 6.3	167.7 ± 5.9
Body mass (kg)	75.5 ± 7.5	60.2 ± 4.7
Body fat (%)	9.6 ± 2.3	18.8 ± 4.6
Weekly training volume (min)	457 ± 204	551 ± 348

**Table 2:** Statistical outcomes showing interaction, time, and sex effects for performance outcomes in the side-hop and pelvic stability tests.

Outcome Measure	Interaction		Time		Sex	
	*p*	$\eta_{p}^{2}$ *, Interpretation*	*p*	$\eta_{p}^{2}$ *, Interpretation*	*p*	$\eta_{p}^{2}$ *, Interpretation*
*Side-hop test*						
Total hops left	0.104	0.054, small	**0.002**	**0.129, medium**	**<0.001**	**0.317, large**
Total hops right	0.319	0.031, small	**<0.001**	**0.228, large**	**<0.001**	**0.324, large**
Valid hops left	0.189	0.227, large	0.148	0.244, large	0.273	0.032, small
Valid hops right	0.191	0.040, small	**0.048**	**0.060, medium**	0.222	0.040, small
Ground contact time left	**0.019**	**0.090, medium**	0.212	0.040, small	**0.016**	**0.146, large**
Ground contact time right	0.225	0.038, small	**0.001**	**0.141, large**	**0.004**	**0.199, large**
*Pelvic stability test*						
Hip drop left supporting	0.788	0.119, medium	0.092	0.354, large	0.828	0.002, no effect
Hip drop right supporting	0.351	0.037, small	0.253	0.045, small	0.858	0.001, no effect

*Note*: bolded values indicate statistically significant medium-to-large effect (*p* < 0.05).

**Table 3:** Mean ± standard deviation performance outcomes in the side-hop and pelvic stability tests across timepoints.

Outcome Measure	Sex	Timepoint						
		T-1	T0	T1	T2	T3	T4	T5
*Side-hop test*								
Total hops left (count)	Males #	30.1 ± 6.3	30.9 ± 2.6	31.5 ± 2.6	31.6 ± 2.3	31.3 ± 3.0	31.2 ± 3.4	30.9 ± 3.6
	Females	26.6 ± 2.1	27.4 ± 2.2	27.5 ± 2.7	28.1 ± 2.3	28.0 ± 2.6	28.4 ± 2.9	28.9 ± 2.7
	Overall	28.4 ± 2.9	29.1 ± 3.0 *	29.4 ± 3.3 *	29.8 ± 2.9 *	29.6 ± 3.3 *	29.8 ± 3.4 *	29.9 ± 3.3 *
Total hops right (count)	Males #	29.8 ± 2.8	31.3 ± 3.1	31.7 ± 2.8	31.8 ± 2.8	31.8 ± 3.3	31.4 ± 3.8	31.7 ± 3.4
	Females	26.2 ± 2.1	27.3 ± 2.0	27.8 ± 2.8	27.7 ± 2.7	27.7 ± 2.9	28.2 ± 3.2	28.9 ± 3.5
	Overall	28.0 ± 3.0	29.2 ± 3.3 *	29.7 ± 3.4 *	29.7 ± 3.4 *	29.7 ± 3.7 *	29.8 ± 3.8 *	30.3 ± 3.7 *
Valid hops left (count)	Males	24.8 ± 3.7	26.1 ± 4.8	25.3 ± 5.4	25.5 ± 5.3	24.9 ± 5.2	24.3 ± 5.2	23.4 ± 5.7
	Females	21.9 ± 3.3	23.4 ± 3.5	23.7 ± 3.0	24.0 ± 3.6	23.5 ± 2.7	24.2 ± 3.5	24.0 ± 2.6
	Overall	23.4 ± 3.8	24.7 ± 4.4	24.5 ± 4.4	24.7 ± 4.5	24.2 ± 4.2	24.2 ± 4.4	23.7 ± 4.4
Valid hops right (count)	Males	24.1 ± 5.3	26.3 ± 4.3	26.2 ± 5.3	25.7 ± 6.0	25.0 ± 5.7	25.1 ± 7.3	24.6 ± 7.2
	Females	22.1 ± 3.4	23.2 ± 3.9	23.4 ± 3.7	23.7 ± 3.3	23.8 ± 3.8	23.5 ± 3.3	24.6 ± 3.2
	Overall	23.1 ± 4.5	24.7 ± 4.4 *	24.7 ± 4.7 *	24.7 ± 4.9	24.4 ± 4.8	24.3 ± 5.7	24.6 ± 5.5
Ground contact time left (s)	Males	0.21 ± 0.02 #	0.20 ± 0.02 #	0.20 ± 0.02 #	0.20 ± 0.02 #	0.21 ± 0.03 #	0.21 ± 0.03	0.21 ± 0.04
	Females	0.23 ± 0.02	0.23 ± 0.02	0.23 ± 0.03	0.23 ± 0.02	0.23 ± 0.03	0.22 ± 0.03	0.22 ± 0.02
	Overall	0.22 ± 0.02	0.21 ± 0.02	0.21 ± 0.03	0.21 ± 0.03	0.22 ± 0.03	0.22 ± 0.03	0.22 ± 0.03
Ground contact time right (s)	Males #	0.21 ± 0.02	0.21 ± 0.02	0.20 ± 0.02	0.20 ± 0.02	0.21 ± 0.03	0.21 ± 0.03	0.21 ± 0.03
	Females	0.24 ± 0.03	0.23 ± 0.02	0.23 ± 0.03	0.23 ± 0.03	0.23 ± 0.03	0.23 ± 0.03	0.23 ± 0.03
	Overall	0.23 ± 0.03	0.22 ± 0.03 *	0.21 ± 0.03 *	0.21 ± 0.03 *	0.22 ± 0.03 *	0.22 ± 0.03 *	0.22 ± 0.03 *
*Pelvic stability test*								
Hip drop left supporting (cm)	Males	31.5 ± 7.2	31.0 ± 6.8	32.4 ± 7.3	33.9 ± 7.3	31.8 ± 10.9	32.0 ± 11.4	31.1 ± 11.0
	Females	28.8 ± 11.7	28.0 ± 11.7	33.5 ± 11.6	32.1 ± 8.9	30.4 ± 10.3	31.6 ± 9.3	30.9 ± 12.6
	Overall	30.1 ± 9.7	29.5 ± 9.5	33.0 ± 9.6	33.0 ± 8.1	31.2 ± 10.5	31.8 ± 10.3	31.0 ± 11.7
Hip drop right supporting (cm)	Males	30.9 ± 9.4	30.9 ± 9.7	32.9 ± 10.0	31.1 ± 9.0	33.4 ± 11.4	31.3 ± 9.6	28.3 ± 10.3
	Females	30.1 ± 8.7	29.4 ± 7.7	33.2 ± 11.6	29.7 ± 8.6	29.7 ± 8.7	27.2 ± 12.3	31.1 ± 10.3
	Overall	30.5 ± 8.9	30.1 ± 8.7	33.1 ± 10.7	30.4 ± 8.7	31.7 ± 10.2	29.3 ± 11.0	29.7 ± 10.3

*Note*: * significantly (*p* < 0.05) different from T-1; # significantly different (*p* < 0.05) to females, either across timepoints (interaction effects presented at each timepoint separately) or overall (main sex effects presented next to “Male”.

## Data Availability

The datasets generated and analyzed in this study are available from the corresponding author on reasonable request.

## Abbreviations

The following abbreviations are used in this manuscript:

ACL	Anterior cruciate ligament
EMG	Electromyography
ICC	Intraclass correlation coefficient
SEM	Standard error of measurement
RPE	Rating of perceived exertion
HR	Hear rate
DBB	Deadbug bridging test
ANOVA	Analyses of variance

## Appendix A

### Appendix A.4. Raw and Processed Electromyography Data from a Representative Participant During Single-Leg Side-Hop and Pelvic Stability Assessments at T1

*Note*: raw (**A**) and processed (**B**) electromyography data during single-leg side-hop assessment; in the single-leg side-hop test, the vertical colored lines indicate successive foot contacts, first recorded on the right plate (Fz1; left plate Fz2), with EMG signals from the corresponding muscles and a total recording duration of 10 s; raw (**C**) and processed (**D**) electromyography data during pelvic stability assessment; in the pelvic stability test red markers indicate a left supporting leg, and green markers indicate a right supporting leg; each side was performed three times (up to 3 s each, from the first event—when the hip marker was at its highest point—to the second event—when the lifted leg was placed back on the force plate), always starting with the left leg.
